# Effect of Intraoperative Dexmedetomidine Use on Postoperative Liver Function and Graft Outcomes in Laparoscopic Living Donor Hepatectomy: A Propensity Score–Matched Study

**DOI:** 10.3390/jcm15051906

**Published:** 2026-03-02

**Authors:** Yan Zhen Jin, Hye-Mee Kwon, Kyoung-Sun Kim, Shi-Yeun Lee, In-Gu Jun, Jun-Gol Song, Gyu-Sam Hwang

**Affiliations:** Department of Anesthesiology and Pain Medicine, Laboratory for Cardiovascular Dynamics, Asan Medical Center, University of Ulsan College of Medicine, 88 Olympic-ro 43-gil, Songpa-gu, Seoul 05505, Republic of Korea; yanzhen9051@163.com (Y.Z.J.); hyemee.kwon@amc.seoul.kr (H.-M.K.); kyoungsun.kim@amc.seoul.kr (K.-S.K.); sophist2036@naver.com (S.-Y.L.); igjun@amc.seoul.kr (I.-G.J.); kshwang@amc.seoul.kr (G.-S.H.)

**Keywords:** dexmedetomidine, graft failure, ischemia–reperfusion injury, laparoscopic living donor hepatectomy, living donor liver transplantation

## Abstract

**Background/Objectives:** Laparoscopic living donor hepatectomy may compromise hepatic microcirculation and exacerbate ischemia–reperfusion injury. Evidence regarding the effects of dexmedetomidine on donor liver injury and graft outcomes in laparoscopic living donor liver transplantation (LDLT) remains limited. **Methods:** We conducted a retrospective cohort study of adult donor–recipient pairs undergoing purely laparoscopic living donor hepatectomy with the Pringle maneuver, categorized by intraoperative dexmedetomidine administration. Primary outcomes were postoperative donor liver function and lactate dynamics. Secondary outcomes included recipient postoperative liver function, perioperative lactate dynamics, early allograft dysfunction (EAD), and graft failure. A 1:1 propensity score matching was performed, and longitudinal laboratory trends were analyzed using linear mixed-effects models. **Results:** Among 395 donor–recipient pairs, 168 matched pairs (84 per group) were analyzed after PSM. Donors receiving dexmedetomidine had significantly lower postoperative peak aspartate aminotransferase (AST) and alanine aminotransferase (ALT) levels (both *p* < 0.01), with significant group-by-time interactions (AST *p* < 0.001; ALT *p* = 0.013). Lactate trajectories differed significantly between groups in both donors and recipients (*p* for interaction < 0.001). In recipients, there were no significant differences between the two groups in EAD (2.4% vs. 8.3%; OR, 0.31; 95% CI, 0.07–1.35; *p* = 0.168) and one-year graft survival (1.2% vs. 4.8%; HR, 0.36; 95% CI, 0.04–7.20; *p* = 0.251). **Conclusions:** Intraoperative dexmedetomidine administration in living liver donors was associated with reduced biochemical evidence of hepatocellular injury and improved perioperative metabolic profiles. These findings suggest a potential donor-level protective effect without demonstrable early clinical benefit in recipients, supporting the need for prospective studies to clarify its clinical significance.

## 1. Introduction

Liver transplantation remains the definitive treatment for end-stage liver disease and certain malignant liver tumors [1,2]. Although advances in perioperative management have improved post-transplant survival rates, early graft dysfunction continues to negatively impact short-term outcomes [3,4]. A growing body of evidence indicates that the donor’s perioperative condition and ischemia–reperfusion injury play critical roles in graft quality and recipient outcomes [5,6]. This highlights the importance of optimizing donor management.

Due to its ability to reduce donor morbidity and facilitate faster recovery, laparoscopic donor hepatectomy is increasingly being adopted in living donor liver transplantation [7,8,9]. However, pneumoperitoneum, the reverse Trendelenburg position, and prolonged hepatic manipulation can compromise venous return. This may lead to hemodynamic instability and disrupt hepatic microcirculation, potentially adversely affecting residual liver function in donors and graft survival [10,11,12,13].

Dexmedetomidine is a highly selective α_2_-adrenergic receptor agonist widely used in perioperative anesthesia due to its sedative and sympatholytic properties [14,15]. Beyond these effects, a growing body of experimental and clinical evidence suggests that dexmedetomidine also exerts anti-inflammatory, antioxidant, and microcirculatory protective effects [16,17,18]. In the context of hepatic surgery and transplantation, dexmedetomidine is believed to attenuate hepatic ischemia–reperfusion injury and preserve hepatocellular function [19,20,21]. These effects are particularly relevant during laparoscopic donor hepatectomy, where pneumoperitoneum-induced reductions in portal venous flow and surgical stress may exacerbate ischemia–reperfusion injury. However, direct clinical evidence supporting these benefits in laparoscopic donor liver retrieval remains limited.

Early postoperative laboratory parameters serve as sensitive indicators of ischemia–reperfusion injury and early graft function and are clinically relevant even in living donor liver transplantation [5,22,23]. However, pharmacologic strategies targeting donor-related perioperative injury during laparoscopic donor hepatectomy remain poorly defined. To date, clinical evidence evaluating dexmedetomidine as a donor-centered intervention to improve graft quality, along with concurrent assessment of early donor and recipient outcomes in laparoscopic living donor liver transplantation, is scarce.

Therefore, this study primarily focused on postoperative laboratory parameters of donors to assess liver function and evaluated the impact of perioperative dexmedetomidine on graft quality and recipient postoperative outcomes following laparoscopic living donor liver resection.

## 2. Materials and Methods

### 2.1. Study Population

We reviewed the electronic medical records of adult living liver donors who underwent laparoscopic living donor liver transplantation (LDLT) at our center from January 2017 to November 2025. Data were censored at the time of IRB approval and database lock.

**Figure 1:**
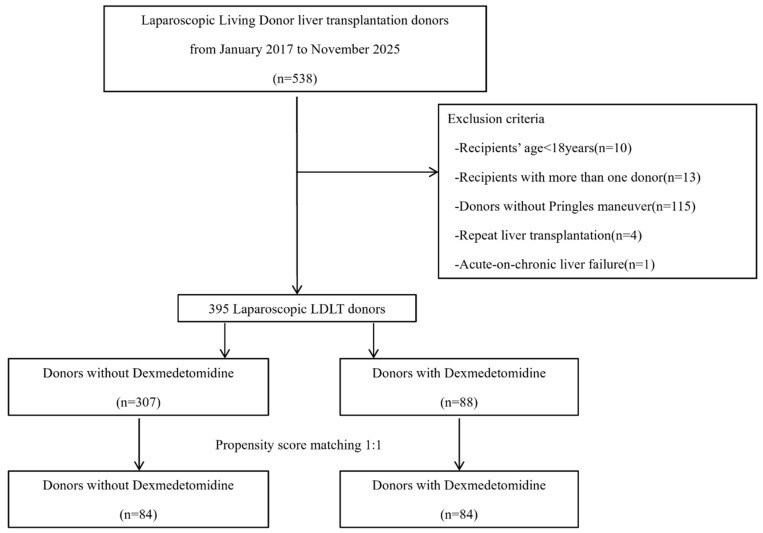
Flowchart of the study population.

The study included adults aged 18 years or older who underwent LDLT. Donor-related exclusion criteria were significant underlying diseases, donor hepatectomy not performed using a purely laparoscopic approach, intraoperative conversion from laparoscopic to open surgery, and absence of the Pringle maneuver during hepatic parenchymal transection. Recipient-related exclusion criteria included age under 18 years, repeat liver transplantation, dual donors, and acute-on-chronic liver failure (Figure 1).

### 2.2. Data Collection and Definitions

Demographic, donor-related, preoperative laboratory, and intraoperative data were collected from the electronic medical records system. Demographic variables included age, sex, body mass index (BMI), and comorbidities such as diabetes mellitus, hypertension, chronic kidney disease, and coronary artery disease. Recipient-related liver disease characteristics included etiology (viral hepatitis, alcoholic hepatitis, biliary disease, and hepatocellular carcinoma), MELD sodium (MELD-Na), and Child–Turcotte–Pugh (CTP) score. Donor-related variables comprised age, sex, total hepatic fatty change, and duration of the Pringle maneuver. Preoperative laboratory data included albumin, prothrombin time–international normalized ratio (INR), total bilirubin (TB), aspartate aminotransferase (AST), and alanine aminotransferase (ALT). Intraoperative variables included anesthetic time, total ischemic time, graft-to-recipient weight ratio (GRWR), fluid administration (crystalloids and 20% albumin), massive transfusion, post-reperfusion syndrome (PRS), and intraoperative epinephrine use. Massive transfusion was defined as the administration of ≥10 units of packed red blood cells (PRBCs) within 24 h or ≥4 units within 1 h [24]. PRS was defined as a decrease in mean arterial pressure of more than 30% from the pre-reperfusion baseline lasting at least 1 min within the first 5 min after graft reperfusion [25].

### 2.3. Anesthetic and Surgical Techniques

All donors and recipients underwent surgery under general anesthesia following a standardized institutional protocol. Anesthesia in donors was induced with propofol, fentanyl, midazolam, and rocuronium, and maintained with desflurane or sevoflurane in combination with opioid analgesics. Dexmedetomidine was administered at the discretion of the attending anesthesiologist, initiated after induction of anesthesia, administered as a continuous infusion at 0.4 μg/kg/h without a loading dose, and discontinued at the end of surgery. Standard monitoring, in accordance with the American Society of Anesthesiologists guidelines, was implemented, including invasive arterial blood pressure monitoring, with central venous catheterization performed as clinically indicated. A goal-directed and relatively restrictive intraoperative fluid management strategy was applied, and vasoactive agents were administered as needed to maintain hemodynamic stability. The general anesthetic principles for recipients were similar to those applied in donors, with continuous invasive hemodynamic monitoring maintained throughout the anhepatic and reperfusion phases. Donors underwent pure laparoscopic donor hepatectomy. During parenchymal transection, intermittent Pringle maneuvers were applied to temporarily occlude hepatic inflow and reduce intraoperative blood loss. Recipient hepatectomy and graft implantation were performed according to standard living donor liver transplantation procedures, followed by vascular anastomoses and biliary reconstruction. Detailed surgical techniques have been described previously [26,27,28].

### 2.4. Study Outcomes

The primary outcome of this study was postoperative liver function laboratory indicators in donors: AST, ALT, TB, and INR. These measurements were recorded at their peak values on postoperative days 1–7 and longitudinal trends over time to assess hepatocellular injury and liver function recovery in donors. The secondary outcomes included short-term postoperative results in recipients, such as liver function laboratory indicators (e.g., AST, ALT, TB, INR) and the incidence of early allograft dysfunction (EAD). Additionally, the study evaluated three-month, six-month, and one-year graft failure rates.

### 2.5. Statistical Analysis

Continuous variables are presented as mean ± standard deviation (SD) or median (interquartile range, IQR), while categorical variables are expressed as counts and percentages. Patients were classified based on whether dexmedetomidine was administered to the donor during laparoscopic living donor liver transplantation.

To balance baseline characteristics between the dexmedetomidine and control groups, propensity scores were estimated using a multivariable logistic regression model, which included recipient characteristics (age, sex, BMI, MELD-Na score, CTP score, diabetes mellitus, hypertension, chronic kidney disease, coronary artery disease, congestive heart failure, viral hepatitis, alcoholic liver disease, hepatocellular carcinoma, and biliary disease), donor characteristics (age, sex, preoperative total fatty change, and Pringle time), GRWR (<0.8), PRS, anesthesia duration, and total ischemia time. One-to-one nearest-neighbor matching without replacement was performed using a caliper of 0.31 on the logit scale of the propensity score. This caliper width was selected based on empirical balance diagnostics and prior methodological recommendations to optimize covariate balance while minimizing sample loss. Covariate balance after matching was assessed using standardized mean differences (SMDs), with an SMD less than 0.1 considered indicative of adequate balance.

Group comparisons were performed using Student’s *t*-test or Mann–Whitney U test for continuous variables, and the Chi-square test or Fisher’s exact test for categorical variables. For categorical outcomes, odds ratios (ORs) with 95% confidence intervals were calculated. Time-to-event outcomes were analyzed using univariable Firth penalized Cox proportional hazards regression to estimate hazard ratios (HRs) with 95% confidence intervals, given the low number of events. In addition, survival probabilities were assessed using the Kaplan–Meier method, and survival curves were compared between groups using the log-rank test. Longitudinal outcomes were analyzed using linear mixed-effects models, including fixed effects for group, time, and their two-way interaction (group × time), with subject-specific random intercepts to account for within-subject correlations arising from repeated measurements.

All statistical analyses were performed using R version 4.5.2 (R Foundation for Statistical Computing, Vienna, Austria). All tests were two-sided, and *p* values less than 0.05 were considered statistically significant.

## 3. Results

### 3.1. Baseline Characteristics Before and After Propensity Score Matching

In the unmatched cohort, 395 donor–recipient pairs were included, comprising 307 donors in the control group and 88 in the dexmedetomidine group. Prior to propensity score matching, multiple baseline characteristics differed between the two groups (Table 1). The dexmedetomidine group had a higher proportion of male donors and a greater prevalence of diabetes mellitus and hypertension. Recipient-related factors, including viral hepatitis, alcoholic hepatitis, and hepatocellular carcinoma, as well as donor-to-recipient weight ratio ≥ 0.8, also showed imbalances between groups. In addition, imbalances were observed in several donor-related, laboratory, and intraoperative variables, with some covariates demonstrating standardized mean differences exceeding 0.2.

After 1:1 propensity score matching, 168 donor–recipient pairs were included, with 84 pairs in each group. Baseline donor demographics, recipient characteristics, donor-related variables, and intraoperative variables were generally well balanced between the dexmedetomidine and control groups. Most post-matching SMDs were below 0.1, indicating improved covariate balance after matching and suggesting that propensity score matching effectively minimized baseline imbalances between the two groups

### 3.2. Postoperative Laboratory Outcomes After Propensity Score Matching

**Figure 2:**
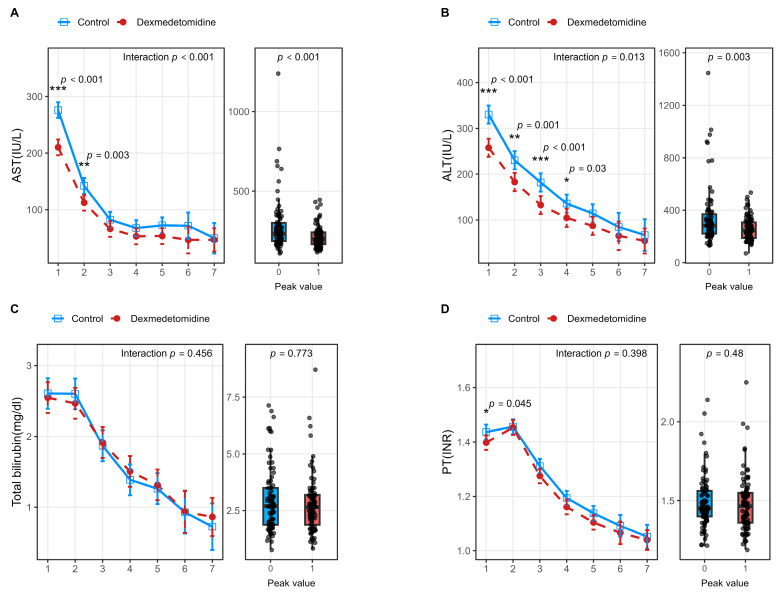
Perioperative laboratory trends and peak values in living liver donors after propensity score matching. Panels (**A**–**D**) depict postoperative trends (line plots) and corresponding peak values (box plots) of donor laboratory parameters, including aspartate aminotransferase (AST) (**A**), alanine aminotransferase (ALT) (**B**), total bilirubin (TB) (**C**), and prothrombin time–international normalized ratio (PT–INR) (**D**). Line plots illustrate serial changes during the postoperative period, while box-and-whisker plots display peak values within 7 postoperative days. Asterisks indicate statistical differences at individual timepoints (* *p*< 0.05, ** *p*< 0.01, *** *p*< 0.001). The *p* for interaction represents the interaction effect between group and time. A two-sided *p* < 0.05 was considered statistically significant. Abbreviations: AST, aspartate aminotransferase; ALT, alanine aminotransferase; INR, international normalized ratio.

**Figure 3:**
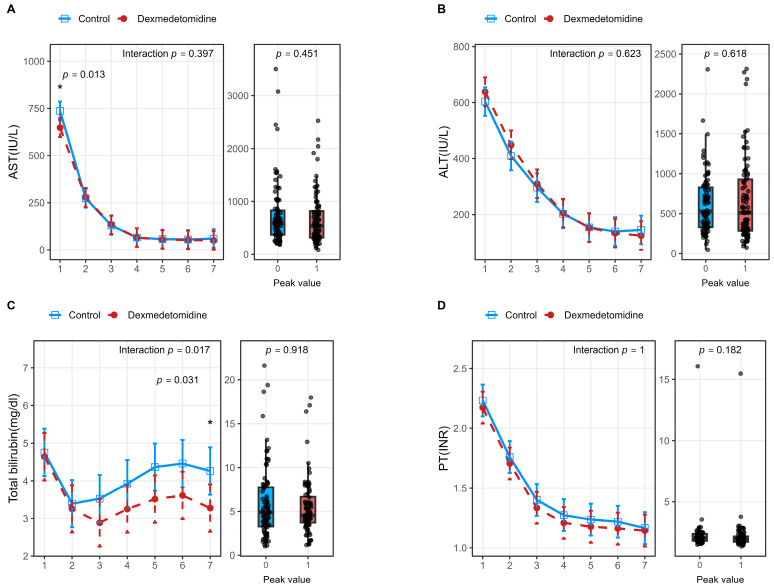
Perioperative laboratory trends and peak values in living liver recipients after propensity score matching. Panels (**A**–**D**) depict postoperative trends (line plots) and corresponding peak values (box plots) of recipient laboratory parameters, including aspartate aminotransferase (AST) (**A**), alanine aminotransferase (ALT) (**B**), total bilirubin (TB) (**C**), and prothrombin time–international normalized ratio (PT–INR) (**D**). Line plots illustrate serial changes during the postoperative period, while box-and-whisker plots display peak values within 7 postoperative days. Asterisks indicate statistical differences at individual timepoints (* *p*< 0.05). The *p* for interaction represents the interaction effect between group and time. A two-sided *p* < 0.05 was considered statistically significant. Abbreviations: AST, aspartate aminotransferase; ALT, alanine aminotransferase; INR, international normalized ratio.

For hepatocellular injury-related markers, postoperative AST and ALT levels in donors exhibited significantly different temporal patterns between the dexmedetomidine and control groups (AST: *p* for interaction < 0.001; ALT: *p* for interaction = 0.013) (Figure 2). Further analysis of peak values demonstrated that donors in the dexmedetomidine group had significantly lower peak AST (*p* < 0.001) and ALT (*p* = 0.003) levels compared with those in the control group (Table 2). In contrast, no significant interaction effects were observed between groups in the temporal trends of postoperative INR or total bilirubin levels. However, in recipients, there were no significant differences observed between groups in the temporal trends of postoperative AST, ALT, or INR levels, and their peak values were also comparable (Figure 3). Although the temporal trend of postoperative total bilirubin levels differed significantly between the two groups (*p* for interaction = 0.017), comparisons of peak values did not reveal a significant difference between groups.

**Figure 4:**
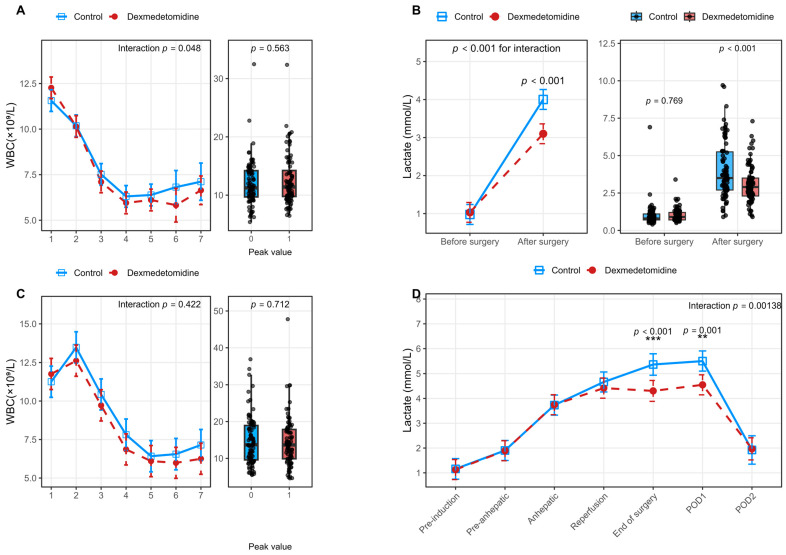
Perioperative WBC (White Blood Cell) and lactate levels in living liver donors and recipients after propensity score matching. (**A**) Description of postoperative changes and peak values of donor WBC. (**B**) Description of perioperative trends and peak values of donor lactate (**C**). Description of postoperative changes and peak values of recipient WBC. (**D**) Description of perioperative trends and peak values of recipient lactate. Line plots illustrate serial changes during the perioperative and postoperative period, while box-and-whisker plots display peak values within 7 postoperative days. Asterisks indicate statistical differences at individual timepoints (** *p*< 0.01, *** *p*< 0.001). The *p* for interaction represents the interaction effect between group and time. A two-sided *p* < 0.05 was considered statistically significant. Abbreviations: WBC, white blood cells.

In addition, the longitudinal changes in donor white blood cell counts differed significantly between the two groups (*p* for interaction = 0.048); however, postoperative peak WBC (white blood cells) values did not differ significantly. As shown in Figure 4, metabolic parameters demonstrated more pronounced group differences, with postoperative lactate trajectories differing significantly between the control and dexmedetomidine groups (*p* for interaction < 0.001). Similarly, in recipients, dynamic postoperative changes in lactate levels showed a significant interaction effect between groups (*p* for interaction < 0.001).

### 3.3. Graft Outcomes Before and After Propensity Score Matching

Among donors, postoperative hospital length of stay did not differ significantly between the dexmedetomidine and Control groups (7 [6–8] days vs. 7 [6,7] days; *p* = 0.622). No donor developed acute kidney injury in either group.

**Figure 5:**
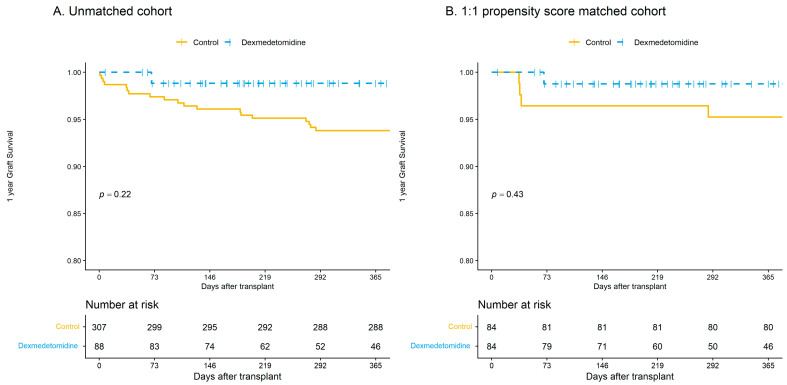
Kaplan–Meier curves for graft survival within one year before and after propensity score matching. Panels (**A**,**B**) illustrate the one-year graft survival trends in the unmatched cohort (*n* = 395) and the 1:1 propensity score-matched cohort (*n* = 168), respectively. Solid yellow lines represent the control group, while dashed blue lines represent the dexmedetomidine group. Vertical ticks on the survival curves indicate censored data. The tables below the plots display the number of recipients at risk at each timepoint. Intergroup comparisons of survival curves were performed using the log-rank test. Two-sided *p* values less than 0.05 were considered statistically significant.

Among recipients in the unmatched cohort (*n* = 395), the incidence of EAD was 0.5% in the dexmedetomidine group and 5.1% in the control group (Table 3). Although the incidence was numerically lower in the dexmedetomidine group, the difference did not reach statistical significance (OR, 0.41; 95% CI, 0.11–1.54; *p* = 0.186). Graft failure within 30, 90, 180, and 365 days after transplantation did not differ significantly between the dexmedetomidine and control groups. Specifically, graft failure within one year occurred in 1.1% of the dexmedetomidine group and 6.2% of the control group. Time-to-event analysis using Cox regression also showed no significant difference in graft failure between groups (HR, 0.30; 95% CI, 0.03–3.27; *p* = 0.094). Kaplan–Meier analysis showed no significant difference in one-year graft survival between groups in the unmatched cohort (log-rank *p* = 0.22) (Figure 5A).

After 1:1 propensity score matching, graft-related outcomes were compared between the dexmedetomidine and control groups in 168 matched patients (84 per group). The incidence of EAD was lower in the dexmedetomidine group than in the control group (2.4% vs. 8.3%), although this difference did not reach statistical significance (OR, 0.31; 95% CI, 0.07–1.35; *p* = 0.168). No significant differences in graft failure were observed between the two groups at 30, 90, 180, or 365 days after transplantation. Graft failure within one year occurred in 1.2% of patients in the dexmedetomidine group and 4.8% in the control group. Cox regression analysis also showed no significant difference in graft failure between groups (HR, 0.36; 95% CI, 0.04–7.20; *p* = 0.251). Kaplan–Meier analysis showed no significant difference in one-year graft survival between groups in the matched cohort (log-rank *p* = 0.43) (Figure 5B).

## 4. Discussion

This study demonstrated that perioperative dexmedetomidine administration in living liver donors was associated with reduced postoperative hepatocellular injury, as reflected by significantly lower AST and ALT levels compared with the control group. In addition, dexmedetomidine use was associated with improved perioperative lactate dynamics in both donors and recipients, suggesting enhanced metabolic stability and tissue perfusion. Despite these donor-level biochemical improvements, recipient early postoperative liver function parameters, including AST, ALT, INR, and peak total bilirubin levels, were comparable between groups, and no significant differences in early allograft dysfunction were observed. Although one-year graft failure occurred less frequently in the dexmedetomidine group, this difference did not reach statistical significance, and effect estimates remained imprecise. Taken together, these findings suggest that intraoperative dexmedetomidine may confer measurable donor-level hepatoprotection without translating into clear early clinical benefits in recipients.

Postoperative transaminase levels in donors typically reflect the extent of surgical trauma, ischemia–reperfusion injury, and perioperative stress responses [29]. Even in healthy living donors, hepatectomy involves extensive parenchymal resection, hemodynamic fluctuations, and physiological alterations related to pneumoperitoneum, all of which may transiently impair hepatic microcirculation and hepatocellular integrity [30]. Accordingly, reductions in postoperative AST and ALT levels observed in the dexmedetomidine group are biologically plausible and consistent with its proposed anti-inflammatory and microcirculatory protective effects. Early liver function in recipients depends on graft quality, ischemia times, recipient disease severity, intraoperative hemodynamics, transfusions, and postoperative management [31]. Therefore, the absence of significant early biochemical differences in recipients should not be interpreted as evidence of no biological effect but rather as a reflection of the multifactorial determinants of early graft function and the limited sensitivity of short-term laboratory markers [31]. Although reductions in transaminase levels reached statistical significance, the magnitude of these changes was modest, and their clinical relevance should be interpreted with caution. Taken together, our findings suggest attenuation of subclinical donor hepatocellular injury rather than a clinically overt improvement in early recipient liver function, consistent with prior perioperative studies in hepatic surgery [18,32].

Perioperative lactate is a sensitive marker of tissue perfusion and metabolic stress, and the observed differences in lactate trajectories in our study may reflect improved perioperative microcirculatory stability associated with dexmedetomidine use rather than a direct indicator of enhanced liver function. Previous studies have reported that dexmedetomidine may enhance hepatic microcirculation by increasing sinusoidal blood flow and reducing focal ischemia, thereby contributing to improved metabolic homeostasis [33,34]. Furthermore, dexmedetomidine was associated with improved postoperative lactate dynamics in recipients, suggesting more favorable systemic perfusion and oxygen utilization during the perioperative period. Because lactate reflects the balance between oxygen delivery and consumption, dynamic changes in lactate levels may reveal early microcirculatory adaptations that precede detectable abnormalities in conventional liver function markers [35]. Accordingly, the lactate findings in this study should be interpreted as supportive physiological evidence of perioperative stabilization rather than as an independent marker of graft functional recovery. Early trends in lactate and modest biochemical changes may reflect subtle modulation of inflammatory and oxidative pathways that potentially contribute to graft resilience [36].

From a clinical perspective, our findings suggest that perioperative dexmedetomidine administration may contribute to improved physiological stability and attenuation of subclinical hepatocellular injury without producing measurable differences in early recipient clinical outcomes. The absence of significant differences in early postoperative endpoints highlights the complexity of graft recovery following transplantation, where outcomes are influenced by multiple perioperative and recipient-related factors beyond donor conditioning alone. Although modest biochemical improvements were observed in the dexmedetomidine group, their clinical implications remain uncertain and should be interpreted cautiously, particularly in the context of low event rates. The limited number of graft failure events may have reduced statistical power to detect small but potentially meaningful differences between groups. In transplantation research, nonsignificant trends may still provide hypothesis-generating signals when supported by biologically plausible mechanisms, including attenuation of perioperative stress and ischemia–reperfusion injury. Accordingly, these findings should be viewed as exploratory rather than confirmatory, and prospective studies with larger cohorts are required to determine whether such perioperative modulation translates into mid- or long-term improvements in graft function [37].

Prior studies in hepatic surgery and liver transplantation have demonstrated that perioperative dexmedetomidine may mitigate ischemia–reperfusion injury and reduce postoperative transaminase elevations, findings that are directionally consistent with the donor-level biochemical improvements observed in the present study [18,21,32,38]. These hepatoprotective effects have been attributed to multiple complementary mechanisms, including sympathetic modulation, attenuation of systemic and local inflammatory responses, reduction in oxidative stress, preservation of hepatic microcirculation, stabilization of mitochondrial function, and decreased hepatocyte apoptosis. Rather than representing isolated pharmacologic effects, these mechanisms collectively support the concept that perioperative dexmedetomidine may function as a form of physiologic conditioning that reduces perioperative cellular stress. The concordance between prior mechanistic evidence and the modest biochemical signals observed in our cohort strengthens the biological plausibility of our findings, while also highlighting that such effects may manifest primarily at a subclinical level.

Beyond its hepatoprotective effects, dexmedetomidine has been reported to improve perioperative hemodynamic stability and metabolic regulation, findings that are consistent with the lactate trajectory differences observed in the present study [18,34,39]. Whereas prior investigations have largely focused on isolated peak postoperative measurements, our study integrates donor- and recipient-level outcomes with dynamic trajectory analyses, highlighting the temporal and context-dependent expression of perioperative pharmacologic effects. Donor-level hepatoprotection may therefore remain clinically unapparent in early recipient laboratory parameters, yet still contribute to adaptive graft responses over time. These observations reinforce the concept that graft resilience arises from multifactorial perioperative influences rather than single biochemical endpoints, and suggest a potential role for donor-directed pharmacologic modulation as an adjunctive perioperative strategy.

In this context, the interpretation of graft recovery should extend beyond biochemical markers alone, as graft structural integrity and hemodynamic adequacy are also critical determinants of functional recovery after transplantation. Future investigations integrating quantitative liver volumetry, venous drainage assessment, and advanced perfusion imaging may provide objective frameworks to evaluate how perioperative pharmacologic interventions influence graft physiology. Such approaches could help clarify the structural–functional correlates of graft resilience and offer mechanistic validation for the subtle biochemical signals observed in the present study. Objective assessments of graft volume, outflow reconstruction adequacy, and microcirculatory perfusion may therefore distinguish pharmacologic modulation of perioperative injury from structural optimization of graft performance [40,41].

Beyond pharmacologic approaches, emerging regenerative strategies—including cell-based therapies, antifibrotic interventions, and bioengineered scaffolds—are being explored to enhance graft perfusion and structural recovery. These modalities target complementary biological pathways such as tissue regeneration, fibrosis attenuation, and vascular remodeling. In this context, perioperative pharmacologic conditioning and regenerative therapies should be considered conceptually complementary rather than competing strategies, as both aim to reduce early injury while promoting adaptive repair processes. Accordingly, integrating pharmacologic modulation with regenerative approaches may represent a future investigational direction rather than an immediate clinical application, potentially contributing to improved graft durability [42,43].

Several limitations should be acknowledged when interpreting the findings of this study. First, dexmedetomidine administration was not protocol-driven but left to the discretion of the attending anesthesiologist, introducing the possibility of confounding by indication. Although propensity score matching was performed to balance measured covariates, residual confounding related to unmeasured perioperative factors cannot be excluded; therefore, causal inferences should be interpreted with caution. Second, this study spans several years (2017–2025), during which surgical proficiency in laparoscopic donor hepatectomy may have evolved. Improvements in surgical technique and perioperative management over time could partially account for the observed associations. Although all procedures were conducted at a high-volume center using standardized protocols, temporal changes and learning-curve effects may have influenced perioperative outcomes. Third, donor clinical outcomes such as pain scores, postoperative nausea and vomiting, delirium, and the Dindo–Clavien classification of surgical complications were not systematically captured in our registry. As a result, the clinical significance of the observed biochemical improvements in donors cannot be fully determined beyond laboratory-based endpoints, limiting the assessment of broader recovery outcomes. Finally, the relatively low number of graft failure events may have limited statistical power and reduced the precision of effect estimates, resulting in wide confidence intervals, increasing the risk of overestimation of treatment effects. Larger prospective cohort studies are required to validate these findings.

## 5. Conclusions

In conclusion, this retrospective propensity score-matched study demonstrated that intraoperative administration of dexmedetomidine in laparoscopic living liver donors was associated with reduced postoperative hepatocellular injury, as reflected by lower transaminase levels, and more favorable perioperative lactate dynamics. However, these biochemical and metabolic indicators did not translate into measurable differences in recipient outcomes.

## Figures and Tables

**Table 1:** Perioperative characteristics of the study population before and after propensity score matching.

		Unmatched (*n* = 395)			Propensity Score Matched (*n* = 168)		
	Total (*n* = 395)	Control (*n* = 307)	Dexmedetomidine (*n* = 88)	SMD	Control (*n* = 84)	Dexmedetomidine (*n* = 84)	SMD
Demographic variables							
Recipient age(years)	56.9 ± 8.81	56.7 ± 8.7	57.2 ± 9.2	0.055	57.8 ± 9.1	57.1 ± 9.3	0.076
Recipient sex, male	255 (64.6)	205 (66.8)	50 (56.8)	0.206	49 (58.3)	49 (58.3)	<0.001
Body mass index (kg/m^2^)	23.3 ± 3.4	23.2 ± 3.2	23.6 ± 3.7	0.112	23.1 ± 3.1	23.5 ± 3.6	0.103
Diabetes mellitus	121 (30.6)	89 (29.0)	32 (36.4)	0.158	31 (36.9)	30 (35.7)	0.025
Hypertension	97 (24.6)	67 (21.8)	30 (34.1)	0.276	26 (31.0)	27 (32.1)	0.026
Chronic kidney disease	17 (4.3)	15 (4.9)	2 (2.3)	0.141	3 (3.6)	2 (2.4)	0.070
Coronary artery disease	22 (5.6)	15 (4.9)	7 (8.0)	0.125	6 (7.1)	6 (7.1)	<0.001
Etiology							
MELD-Na score	12.9 ± 6.1	12.7 ± 6.3	13.4 ± 5.6	0.115	13.1 ± 6.4	13.5 ± 5.7	0.055
Child-Pugh-Turcotte score	7.5 ± 2.1	7.4 ± 2.1	7.9 ± 2.1	0.194	7.7 ± 2.2	7.9 ± 2.1	0.092
Viral hepatitis	216 (54.7)	180 (58.6)	36 (40.9)	0.360	38 (45.2)	36 (42.9)	0.048
Alcoholic hepatitis	128 (32.4)	89 (29.0)	39 (44.3)	0.322	33 (39.3)	36 (42.9)	0.073
Biliary disease	24 (6.1)	18 (5.9)	6 (6.8)	0.039	8 (9.5)	6 (7.1)	0.086
Hepatocellular carcinoma	197 (49.9)	163 (53.1)	34 (38.6)	0.293	34 (40.5)	34 (40.5)	<0.001
GRWR ≥ 0.8	347 (87.8)	264 (86.0)	83 (94.3)	0.282	79 (94.0)	79 (94.0)	<0.001
Donor-related variables							
Donor age (years)	29.3 ± 7.6	28.7 ± 7.1	31.2 ± 9.0	0.303	30.7 ± 7.0	31.1 ± 9.1	0.051
Donor sex, male	193 (48.9)	140 (45.6)	53 (60.2)	0.296	46 (54.8)	49 (58.3)	0.072
Total fatty change (%)	3.5 ± 4.4	3.3 ± 4.5	4.1 ± 4.3	0.175	3.9 ± 4.5	4.0 ± 4.4	0.040
Pringle maneuver time (min)	93.6 ± 32.1	95.9 ± 32.8	85.4 ± 28.3	0.344	85.7 ± 29.6	85.2 ± 28.8	0.018
Preoperative laboratory data							
Albumin (g/dL)	3.2 ± 0.6	3.2 ± 0.6	3.2 ± 0.5	0.012	3.1 ± 0.6	3.1 ± 0.5	0.061
INR	1.3 ± 0.3	1.3 ± 0.3	1.4 ± 0.3	0.077	1.3 ± 0.3	1.4 ± 0.3	0.053
Total bilirubin (mg/dL)	2.5 ± 4.0	2.4 ± 4.1	2.8 ± 3.4	0.097	2.8 ± 4.6	2.9 ± 3.4	0.011
AST (IU/L)	41.1 ± 34.4	39.3 ± 25.3	47.3 ± 55.4	0.185	37.4 ± 25.6	48.4 ± 56.5	0.250
ALT (IU/L)	27.8 ± 52.2	24.4 ± 18.9	39.6 ± 104.4	0.202	20.3 ± 16.3	40.8 ± 106.8	0.268
Intraoperative variables							
Anesthetic time(hour)	13.4 ± 1.6	13.6 ± 1.6	12.8 ± 1.2	0.598	12.7 ± 1.4	12.8 ± 1.2	0.083
Total ischemic time (min)	128.1 ± 27.3	127.1 ± 26.6	131.4 ± 29.6	0.152	132.2 ± 29.9	130.9 ± 30.0	0.042
Crystalloid (ml)	7148.2 ± 3279.4	7228.0 ± 3234.8	6869.7 ± 3434.7	0.107	6701.8 ± 2594.7	6976.6 ± 3458.4	0.090
20% albumin (ml)	887.1 ± 759.7	883.1 ± 669.3	901.1 ± 1018.8	0.021	846.0 ± 508.1	917.9 ± 1039.6	0.088
Massive transfusion	114 (28.9)	88 (28.7)	26 (29.5)	0.019	22 (26.2)	25 (29.8)	0.080
Post-reperfusion syndrome	343 (86.8)	261 (85.0)	82 (93.2)	0.264	77 (91.7)	78 (92.9)	0.045
Recipient Epinephrine use	103 (26.3)	77 (25.2)	26 (29.9)	0.104	22 (26.5)	23 (27.7)	0.027
Donor Ephedrine use	95 (24.1)	74 (24.1)	21 (23.9)	0.006	18 (21.4)	20 (23.8)	0.057

Continuous data are shown as means ± SD, and categorical data are presented as numbers and percentages; SMD, standardized mean difference. An SMD < 0.1 was considered indicative of adequate balance; Abbreviations: ALT, alanine aminotransferase; AST, aspartate aminotransferase; GRWR, Graft-to-Recipient weight ratio; INR, international normalized ratio; MELD-Na score, Model for End-Stage Liver Disease sodium score.

**Table 2:** Postoperative laboratory parameters within 7 days after liver transplantation.

	Control (*n* = 84)	Dexmedetomidine (*n* = 84)	Total (*n* = 168)	*p*
Donor-related laboratory data				
AST maximum value	231.0 (185.5–300.3)	197.5 (165.5–239.8)	213.0 (174.8–265.5)	<0.001
ALT maximum value	283.5 (221.0–369.3)	245.5 (187.5–307.8)	262.0 (207.0–328.0)	0.003
INR maximum value	1.45 (1.40–1.56)	1.46 (1.36–1.55)	1.46 (1.37–1.56)	0.480
Total bilirubin maximum value	2.70 (1.87–3.50)	2.65 (1.87–3.20)	2.70 (1.87–3.30)	0.773
Recipient-related laboratory data				
AST maximum value	584.0 (370.8–831.8)	538.00 (318.0–820.5)	562.00 (326.0–831.8)	0.451
ALT maximum value	530.0 (330.3–826.5)	511.00 (285.3–929.5)	517.50 (307.3–855.8)	0.619
INR maximum value	2.04 (1.81–2.37)	1.96 (1.72–2.16)	1.99 (1.79–2.29)	0.182
Total bilirubin maximum value	4.90 (3.27–7.75)	4.55 (3.72–6.67)	4.65 (3.50–7.35)	0.918

Data are presented as median (interquartile range); Abbreviations: ALT, alanine aminotransferase; AST, aspartate aminotransferase; INR, international normalized ratio.

**Table 3:** Recipient-related postoperative outcomes before and after propensity score matching.

		Unmatched (*n* = 395)				Matched (*n* = 168)			
Outcome	Time	Control (%)	Dex (%)	OR or HR (95% CI)	*p*	Control (%)	Dex (%)	OR or HR (95% CI)	*p*
EAD	–	20 (5.1)	2 (0.5)	0.41 (0.11–1.54)	0.186	7 (8.3)	2 (2.4)	0.31 (0.07–1.35)	0.168
Graft failure	30 days	4 (1.3)	0 (0)	0.39 (0.01–37.05)	0.469	0 (0)	0 (0)	1.01 (0.11–3.93)	0.938
	90 days	9 (2.9)	1 (1.1)	0.55 (0.06–10.94)	0.469	3 (3.6)	1 (1.2)	0.43 (0.04–13.52)	0.364
	180 days	12 (3.9)	1 (1.1)	0.43 (0.05–5.82)	0.271	3 (3.6)	1 (1.2)	0.43 (0.04–13.52)	0.364
	365 days	19 (6.2)	1 (1.1)	0.30 (0.03–3.27)	0.094	4 (4.8)	1 (1.2)	0.36 (0.04–7.20)	0.251

Values are presented as *n* (%). Odds ratios (ORs) with 95% confidence intervals (CIs) were estimated using logistic regression for early allograft dysfunction (EAD). Univariable Firth penalized Cox proportional hazards regression was used to estimate hazard ratios (HRs) with 95% CIs. Abbreviations: Dex, dexmedetomidine; CI, confidence interval; OR, odds ratio; HR, hazard ratio; EAD, early allograft dysfunction.

## Data Availability

The data presented in this study are available from the corresponding author upon reasonable request. The data are not publicly available due to ethical and privacy restrictions.

## Abbreviations

The following abbreviations are used in this manuscript:

AST	Aspartate Aminotransferase
ALT	Alanine Aminotransferase
BMI	Body Mass Index
CI	Confidence Interval
CTP	Child–Turcotte–Pugh
EAD	Early Allograft Dysfunction
GRWR	Graft-to-Recipient Weight Ratio
HR	Hazard Ratio
INR	International Normalized Ratio
IQR	Interquartile Range
I/R	Ischemia–Reperfusion
LDLT	Living Donor Liver Transplantation
MELD-Na	Model for End-Stage Liver Disease Sodium
OR	Odds Ratio
PRS	Post-Reperfusion Syndrome
PSM	Propensity Score Matching
SD	Standard Deviation
TB	Total Bilirubin
WBC	White Blood Cell
